# Antimicrobial Compounds From *Aspergillus chevalieri* Associated With the Gut Microbiota of *Hermetia illucens* Larvae Targeting *Salmonella enterica* Serovar Pullorum

**DOI:** 10.1155/ijm/8756981

**Published:** 2025-10-15

**Authors:** Mario Ruiz, Billy Cabanillas, Mohamed Haddad, Alvaro Díaz, Michel Sauvain, Denis Castillo

**Affiliations:** ^1^Laboratorios de Investigación y Desarrollo, Facultad de Ciencias e Ingeniería, Universidad Peruana Cayetano Heredia, Lima, Peru; ^2^UMR 152 PharmaDev, Institut de Recherche pour le Développement (IRD), Université de Toulouse (UT), Toulouse, France

**Keywords:** antimicrobial activity, *Aspergillus chevalieri*, black soldier fly, *Hermetia illucens*, natural products, *Salmonella enterica* serovar Pullorum, secondary metabolites

## Abstract

**Background:**

The gut microbiota of insects represents an underexplored reservoir of bioactive compounds with potential antimicrobial applications. This study is aimed at identifying antimicrobial compounds from fungi associated with the gut microbiota of *Hermetia illucens* (black soldier fly) larvae active against *Salmonella enterica* serovar Pullorum, an important poultry pathogen.

**Methods:**

Fungi isolated from *H. illucens* larval gut were screened for antimicrobial activity against *S. enterica* serovar Pullorum. The active fungus was identified through molecular analysis of ITS, benA, and CaM regions. Ethyl acetate extract from the fungal culture was assessed for antimicrobial activity, followed by bioguided fractionation using preparative and semipreparative chromatography. Active fractions were analyzed using UHPLC/HRMS, and putative compound identification was performed through mass spectrometry and molecular networking.

**Result:**

*Aspergillus chevalieri* was identified as active against *S. enterica* serovar Pullorum, with the ethyl acetate extract exhibiting moderate antimicrobial activity (MIC = 4.00 mg/mL; IC_50_ = 3.00 ± 0.58 mg/mL). Bioguided fractionation resulted in several active fractions. UHPLC/HRMS analysis putatively annotated 10 compounds, previously shown to be bioactive, including diketopiperazines (Neoechinulin A and echinulin), peptide derivatives (cyclo(L-Tyr-L-Pro) and *N*-acetyltyramine), a benzofuran derivative, an isocoumarin (diaporthin), a flavin (lumichrome), an aminopurine (isopentenyladenine), and two diterpenoids (4-deoxyphorbol-13-acetate and austinoneol).

**Conclusion:**

This study represents the first report of *A. chevalieri* associated with *H. illucens* larvae and demonstrates its potential as a source of antimicrobial compounds against *S. enterica* serovar Pullorum. The identified bioactive compounds provide promising leads for the development of new antimicrobial agents for poultry health.

## 1. Introduction

The gut microbiota of insects represents a rich source of bioactive compounds with potential applications against various pathogens [1, 2]. Among these, the microbiota of black soldier fly (BSF) larvae, namely, *Hermetia illucens* (*H. illucens*), has gained attention for its antimicrobial properties [3, 4]. BSF larvae are recognized for their ability to efficiently convert organic waste into nutrient-rich biomass for feed supplements [5] while harboring a diverse microbiome capable of inhibiting harmful pathogens associated with organic waste decomposition [6].

While metagenomic profiling and the bioactive potential of *H. illucens* larval microbiota have garnered significant interest [7], the targeted application of these compounds against specific pathogens remains largely unexplored. *Salmonella enterica* is a Gram-negative rod-shaped bacterium and a leading cause of global foodborne illness, representing a prime candidate for such investigation. Typically transmitted via contaminated animal products, especially poultry [8], this bacterium belongs to the Enterobacteriaceae family, is motile via flagella, and exhibits facultative anaerobic metabolism, which allows survivability among a wide range of environmental conditions [9]. This species is taxonomically diverse, comprising more than 2600 serovars classified by surface antigens [10]. Among *S. enterica* serovars, *Salmonella enterica* serovar Pullorum (*S.* Pullorum) is a poultry-specific pathogen and the causative agent of pullorum disease, which is associated with severe economic consequences in poultry farming due to high chick mortality and diminished egg production [11, 12]. Given its documented impact on poultry health and its ongoing relevance to veterinary medicine and food safety [12–15], *S*. Pullorum was selected as the target pathogen for this study.

The overuse of antibiotics to control *S.* Pullorum has contributed to increased bacterial resistance, threatening both poultry health and human health through contaminated meat and environmental waste [16, 17]. This highlights the urgent need to discover new sustainable antimicrobial strategies to manage this pathogen effectively.

*Aspergillus chevalieri* (*A. chevalieri*), formerly *Eurotium chevalieri*, is a xerophilic, filamentous fungus that plays an important role as a decomposer in low-moisture environments such as stored animal feed, dry food products, and dry animal substrate [18–20]. This species is characterized by its ability to grow in low water activity (aw < 0.90) environments, enabling it to colonize substrates that are too dry for other microorganisms. For example, *A. chevalieri* is frequently found in rabbit, chinchilla, and poultry feed, indicating its adaptation to nutrient-rich but moisture-limited ecological niches [18, 20].

Morphologically, *A. chevalieri* exhibits both sexual and asexual reproduction. In its asexual phase, it forms conidiophores, a specialized structure with globose vesicles, from which the conidia arise [18, 21]. In its sexual phase, the fungus produces cleistothecia comprising ascospores, which contribute to long-term environmental survival [19, 22].

*A. chevalieri is* also known for its notable capacity to produce a variety of secondary metabolites with antibacterial properties, for example, indole diketopiperazine alkaloids [23] and cladosporin [18], many of which are associated with toxic effects. In addition, other secondary metabolites, such as Neoechinulin A/B or echinulin, have been identified as taxonomic markers [19] and may also confer antibacterial, antifungal, and anticancer bioactivities [18, 24, 25].

Although *A. chevalieri* has been studied as a contaminant of stored feeds [19], its potential role within the microbial community of *H. illucens* has not been previously explored [6, 26]. While this fungus has not yet been isolated from the gut microbiota of *H. illucens*, its consistent presence in feed substrates and its ability to produce a wide range of antimicrobial secondary metabolites suggest a commensal or mutualistic role, contributing to a microbial balance and defense in *H. illucens* gut [18, 27]. Given its metabolic potential and ecological association with feed-related niches, *A. chevalieri* merits investigation as a contributor to the antimicrobial pool of the BSF larval microbiota, particularly regarding its antimicrobial potential against pathogens such as *S.* Pullorum.

This study is aimed at exploring the gut microbiota of *H. illucens* larvae as a novel source of antimicrobial compounds, specifically focusing on the identification and characterization of bioactive metabolites from *A. chevalieri* effective against *S.* Pullorum. This approach represents a promising avenue for discovering new antimicrobial agents with applications in poultry health and potentially human medicine.

## 2. Materials and Methods

### 2.1. Microorganisms

Filamentous fungi from the digestive tract of *H. illucens* were isolated in a previous study. Briefly, larvae were collected in triplicate from independent breeding cycles. Ten larvae per group were surface-sterilized using 70% ethanol, followed by 15 min of UV exposure. Each larva was dissected under sterile conditions; midgut portions (~0.5 cm) were pooled, suspended in 200 *μ*L of 0.89% saline solution, and serially diluted (10^−1^ to 10^−4^); and 10 *μ*L of each dilution was seeded onto potato dextrose agar (PDA) or Sabouraud dextrose agar (SDA) (BD Difco, Sparks, Maryland, United States), both supplemented with chloramphenicol (100 mg/L) and gentamicin (50 mg/L). To monitor external contamination, controls consisting of the diluent and surface swabs from disinfected larvae were processed in parallel. Plates were incubated at 30°C ± 1°C for 21 days. Fungal colonies with distinct morphologies were subcultured repeatedly to obtain pure isolates [7].

For the present study, these previously isolated fungal strains were reactivated on SDA and incubated at 30°C for 7 days prior to antimicrobial testing.

The bioactivity testing against *S.* Pullorum ATCC 13036 was performed exclusively in the present study. The strain was reactivated from a lyophilized vial (Microbiologics) on trypticase soy agar (TSA) (BD BBL, Sparks, Maryland, United States), incubated at 37°C for 18 h, and subsequently cryopreserved at −80°C in 1.5-mL cryovials containing tryptone soya broth (HiMedia, Maharashtra, India) supplemented with 50% glycerol.

All antimicrobial assays described in this study—including agar well diffusion (Section 2.2), broth microdilution (Section 2.6), and agar overlay bioautography (Section 2.7)—were conducted exclusively against *S*. Pullorum ATCC 13036. No other *Salmonella* serovars were screened. The decision to focus on *S*. Pullorum was based on literature highlighting its persistent importance as a poultry-specific pathogen with high mortality rates in young chickens, significant economic impact, and relevance in food safety and veterinary health management, particularly in low- and middle-income countries [13, 28].

### 2.2. Antibacterial Activity Assay of Filamentous Fungi vs. *S.* Pullorum

Antagonism tests were performed using the agar well technique [29]. After a 7-day incubation period to induce sporulation, fungal spores were released by adding 20 mL of sterile 0.01% Tween 80 (Sigma-Aldrich, Saint Louis, Missouri, United States) to the culture. The spores were quantified and adjusted to a concentration of 10^3^ spores/mL in sterile saline solution [30]. A 10 *μ*L aliquot of this spore suspension was inoculated into wells on sterile SDA plates and incubated at 30°C for 7 days. Subsequently, an 18-h culture of *S.* Pullorum adjusted to 10^6^ CFU/mL (in a Neubauer chamber) in tryptone soya broth was inoculated around the fungal growth area. The plates were incubated at 37°C for 24 h to observe inhibition halos. Tetracycline (0.5 mg/mL; Sigma-Aldrich, Saint Louis, Missouri, United States) served as a positive control. All experiments were performed in triplicate.

### 2.3. Strain Identification of Active Fungus

A pure culture of the active fungus (HGU11_3) was sent to Macrogen (Seoul, Korea) for identification via ITS region amplification using primers ITS5 and ITS4. These primers targeted the ITS1, 5.8S, ITS2, and LSU rRNA regions. Genomic DNA was also extracted from the fungal isolate, and the *β*-tubulin (benA) and calmodulin (CaM) genes were amplified using primers bt2a and bt2b [31] and cmd5 and cmd6 [32], respectively. This analysis was conducted by BTS Consultores (Lima, Peru). The primer sequences are listed in Table 1.

The ITS, benA, and CaM sequences were compared to GenBank data using BLASTn analysis [33]. The ITS sequence was matched against the rRNA/ITS database, while the benA and CaM sequences were compared against the standard nucleotide database. DNA sequences were submitted to GenBank. Additionally, the morphological characteristics of the isolate were evaluated on SDA and under light microscopy using lactophenol cotton blue staining. Colony morphology was documented after 10 days of incubation at 30°C, and microscopic features of the conidiophores were observed. To assess its osmotolerance, the strain was also cultured in triplicate on SDA supplemented with 25% (*w*/*v*) glucose and 15% (*w*/*v*) NaCl under the same incubation conditions.

### 2.4. Phylogenetic Analysis

For phylogenetic analysis, the ITS, benA, and CaM sequences were aligned using MAFFT V7.520 with the L-INS-i strategy and concatenated into a single multilocus dataset using MEGA X. The concatenated dataset was analyzed in IQ-TREE with the TNe + I evolutionary model, selected by the Bayesian information criterion (BIC). A maximum likelihood tree was constructed with 1000 ultrafast bootstrap replicates, with *Penicillium chrysogenum* as an outgroup. The tree was visualized using MEGA X, with bootstrap values and taxon labels added for interpretation.

### 2.5. Preparation of Active Extract From Fungal Culture

A conidial suspension of the identified fungus adjusted to 5 × 10^4^ spores/mL was inoculated into 100 mL of Sabouraud broth (SBB) (BD Difco, Sparks, Maryland, United States) and incubated at 30°C with agitation at 150 rpm for 3 days. The resulting culture was scaled up to 10 L following successive transfers into larger volumes of media. The mycelium was separated from the broth by vacuum filtration. The broth was extracted three times with ethyl acetate (EtOAc) at 1:1 *v*/*v* (JT Baker, Radnor, Pennsylvania, United States). The combined organic phases were concentrated by a rotary evaporator (Büchi R-114, Flawil, Switzerland), yielding 1.853 g of EtOAc crude extract.

### 2.6. Antimicrobial Activity Assay

The broth microdilution method [34] was employed to determine the half-maximal inhibitory concentration (IC_50_) and minimum inhibitory concentration (MIC) of the EtOAc crude extract. Final EtOAc crude extract concentrations ranged from 16 to 0.03 mg/mL, with DMSO (Sigma-Aldrich, Saint Louis, Missouri, United States) maintained below 1%. After 24 h of incubation at 37°C, optical density (OD) was measured at 595 nm. Tetracycline served as a positive control at concentrations ranging from 8 to 0.015 *μ*g/mL. Dose–response curves were fitted using four-parameter logistic regression in GraphPad Prism 8. The MIC was determined visually as the lowest concentration without observable turbidity. All analyses were performed in triplicate.

### 2.7. Agar Overlay Bioautography Assay

The EtOAc crude extract was evaluated using the agar overlay bioautography assay [35]. Spots of 200 *μ*g of extract and 0.5 *μ*g of tetracycline were deposited onto aluminum-backed silica gel 60 F_264_ plates (Merck, Darmstadt, Germany). Plates were UV-sterilized, overlaid with Müller–Hinton agar (BD Difco, Sparks, Maryland, United States), and seeded with *S.* Pullorum at a concentration of 1 × 10^7^ CFU/mL. After incubation, bacterial growth was revealed by spraying a 5 mg/mL 3-(4,5-dimethylthiazol-2-yl)-2,5-diphenyltetrazolium bromide solution (MTT) (Sigma-Aldrich, Saint Louis, Missouri, United States). Inhibition zones appeared as yellow areas against a purple background. This method was also applied to evaluate fractions and subfractions obtained from chromatographic separations.

### 2.8. Isolation and Identification of Compounds

The EtOAc crude extract (1.8533 g) was fractionated on silica gel 60 (0.015–0.040 mm; Merck, Darmstadt, Germany) using a MPLC column and a solvent pump (Büchi, Flawil, Switzerland) with a dichloromethane-to-methanol gradient (*v*/*v*, 0:1 to 1:0; JT Baker, Radnor, Pennsylvania, United States), yielding 15 fractions (F1–F15). Masses for all fractions are reported in Table S5; in brief, the active fractions yielded the following amounts: F6 = 117.5 mg, F9 = 316.3 mg, and F10 = 149.4 mg.

Active fractions (F6, F9, and F10) were further purified using puriFlash XS 520 Plus equipment (Interchim, Montluçon, France) with a DCM-to-MeOH gradient (*v*/*v*, 0:1 to 1:0), producing Subfractions F6.1–F6.11, F9.1–F9.15, and F10.1–F10.9. Individual subfraction masses are provided in Table S6.

### 2.9. UHPLC/HRMS Analysis

Chromatographic analyses were conducted on an UltiMate 3000 UHPLC system (Thermo Scientific, Dreieich, Germany) equipped with a photodiode array detector, column oven, and autosampler. A Luna Omega C18 column (150 × 2.1 mm, 1.6 *μ*m; Phenomenex, Torrance, California, United States) was used at 40°C, with a flow rate of 0.25 mL/min and an injection volume of 2 *μ*L. Mobile phases consisted of 0.1% formic acid in water (A) and 0.1% formic acid in acetonitrile (B). Gradient elution was as follows: 10% B (0–1.5 min), 10%–95% B (1.5–20 min), 95% B (20–21 min), 95%–10% B (21–23 min), and 10% B (23–30min). An equilibration time of 10 min was applied between injections.

Spectrometric data were acquired using a Q Exactive Plus mass spectrometer (Thermo Scientific, Dreieich, Germany) in positive mode, equipped with a heated electrospray ionization source (HESI) and an Orbitrap analyzer. The HESI parameters were set as follows: capillary temperature, 300°C; spray voltage, 3.50 kV; sheath gas and auxiliary gas flow rates, 50 and 10 units, respectively; and S-lens RF level, 70. Full mass spectra were acquired at a resolution of 70,000 (full width at half maximum [FWHM] at *m*/*z* 400) from 120 to 1600 *m*/*z*. High-energy collision-induced dissociation (HCD) was operated at 20, 40, and 60 eV.

### 2.10. Data Processing and Molecular Networking

Data were recorded with Xcalibur software (Version 4.0.27.19) and processed with MzMine3 software [36]. A molecular network was generated using feature-based molecular networking [37] in GNPS (Global Natural Products Social Molecular Networking) with the available spectral databases to generate putative annotations of chemical compounds. All identifications presented in this study are considered Level 2 (putatively annotated compounds) according to the Metabolomics Standards Initiative (MSI) guidelines [38]. Visualizations were created using Cytoscape Version 3.10.1 [39].

## 3. Results

Only one *Salmonella* strain, *S.* Pullorum (ATCC 13036), was tested throughout this study. Antimicrobial assays, including agar well diffusion, broth microdilution, and agar overlay bioautography, were conducted solely against this strain. Therefore, the observed antimicrobial activity of *A. chevalieri* and its derived fractions must be interpreted as specific to *S*. Pullorum under the tested conditions. No other *Salmonella* serovars were included in the screening panel.

### 3.1. Identification of Active Fungus

**Figure 1:**
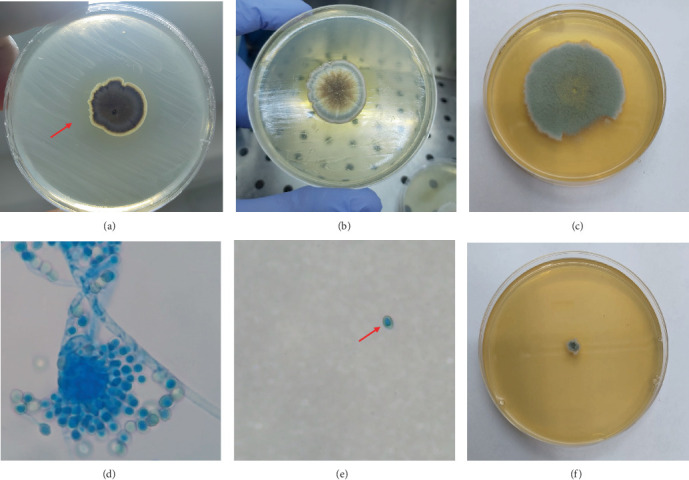
(a) Antagonism test of *A. chevalieri* HGU11_3 against *S*. Pullorum. The red arrow indicates the inhibition halo caused by *A. chevalieri* against *S*. Pullorum. (b) Colony on SDA after 10 days at 30°C, showing a velvety/floccose texture with a brown–gray center and pale margin. (c) Colony growth on SDA supplemented with 25% glucose. (d) Conidiophore with smooth stipe and globose vesicle bearing a compact head of phialides and chains of conidia (lactophenol cotton blue, 400×). (e) Individual subglobose conidia (lactophenol cotton blue, 400×). (f) Colony growth on SDA supplemented with 15% NaCl.

From the fungi isolated from the gut microbiota of *H. illucens* [7], antagonism tests revealed that the filamentous fungal strain HGU11_3 showed activity against *S.* Pullorum (Figure 1a). Molecular identification based on the ITS, benA, and CaM markers showed 100% sequence identity with *A. chevalieri*, according to BLASTn searches (Tables S1–S3). The accession numbers for each molecular marker of *A. chevalieri* HGU11_3 are listed in Table 2.

#### 3.1.1. Colony Characteristics

Colonies grown on SDA developed a velvety to floccose colony, reaching 2.5 to 3 cm after 10 days at 30°C. They exhibited a brown–gray central zone and a whitish to pale greenish margin; the colony edge was well defined with slight undulations, and the reverse appeared yellowish-ochre (Figure 1b). Under osmotic stress conditions, growth was markedly affected: On SDA supplemented with 25% glucose, colonies expanded to ≈4.9 cm, while on SDA with 15% NaCl, growth was severely restricted (≈0.6 cm) (Figure 1e,f).

#### 3.1.2. Micromorphology

Light microscopy with lactophenol cotton blue revealed typical *Aspergillus* features: hyaline, smooth conidiophores terminating in globose vesicles bearing a compact, radially arranged head of phialides, and producing chains of conidia (Figure 1). Conidia measured 3.2–6 *μ*m × 2.7–5.1 *μ*m were globose to subglobose and ranged from smooth to finely roughened (Figure 1). These characteristics are consistent with descriptions of *A. chevalieri* reported in the literature [19, 21] and support its molecular identification.

**Figure 2:**
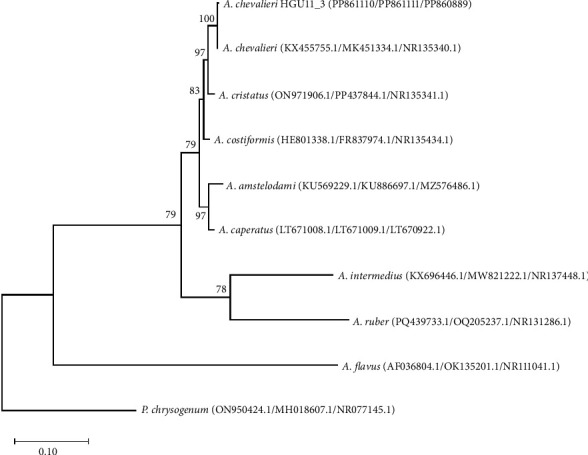
Phylogenetic tree based on the concatenated sequences of the *β*-tubulin (benA), calmodulin (CaM), and ITS region genes, showing the position of *A. chevalieri* HGU11_3 with respect to closely related species. The phylogenetic tree was constructed using the maximum likelihood method provided within the IQ-TREE software Version 2.0.7. Numbers at the nodes indicate percentages of bootstrap support, derived from 1000 ultrafast bootstrap replicates. The numbers in parentheses are GenBank accession numbers in the order of benA/CaM/ITS. Penicillium chrysogenum (ON950424.1/MH018607.1/NR077145.1) was used as an outgroup in these analyses. Bar: patristic distance of 0.10.

Phylogenetic analysis (Figure 2) based on the concatenated sequences of the benA, CaM, and ITS markers successfully resolved the taxonomic identity of the fungal isolate *A. chevalieri* HGU11_3. The isolate clustered unequivocally within the clade of *A. chevalieri* (KX455755.1/MK451334.1/NR135340.1), supported by a bootstrap value of 100, confirming its species identity with high confidence. The inclusion of *P. chrysogenum* as an outgroup effectively rooted the tree and validated its phylogenetic structure, distinctly positioning the *Aspergillus* clades.

### 3.2. Antimicrobial Activity of *A. chevalieri* EtOAc Crude Extract

**Figure 3:**
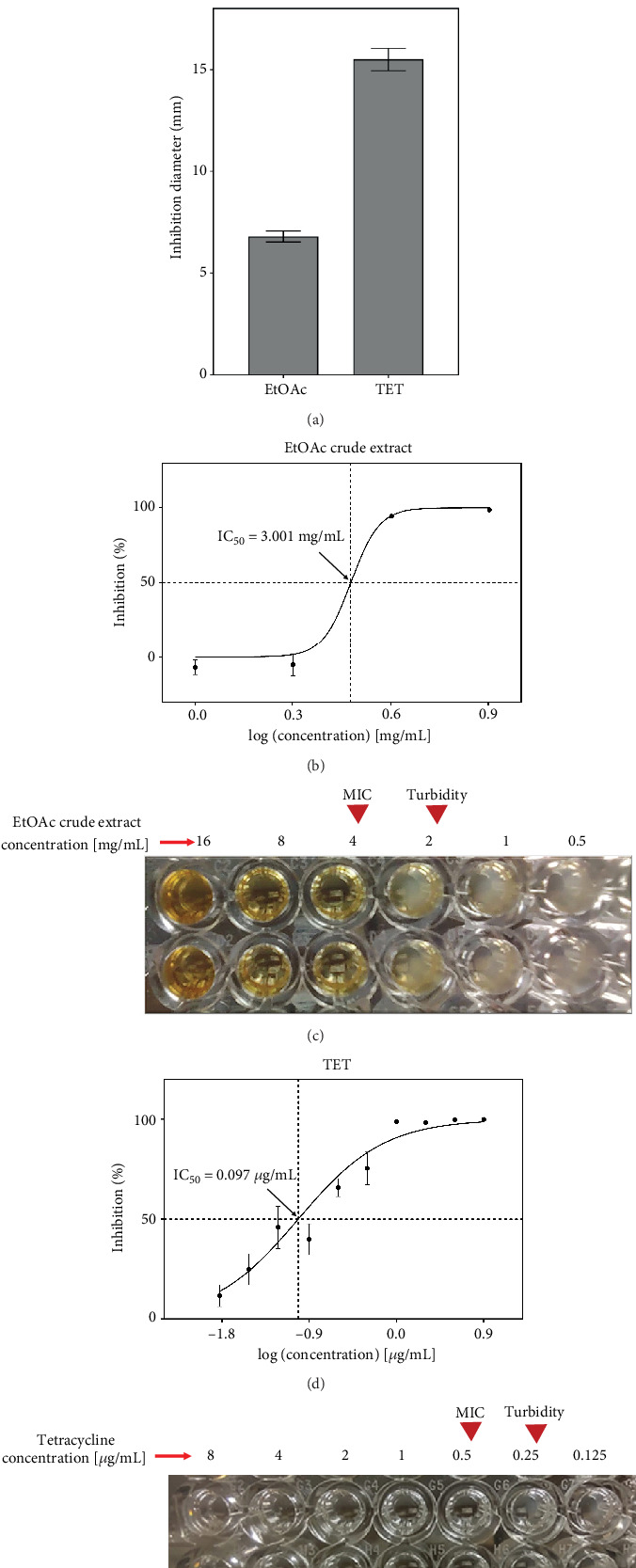
Antimicrobial assays of the EtOAc crude extract from *A. chevalieri* HGU11_3. (a) Bioautography and inhibition halos of the EtOAc crude extract and tetracycline (TET). SC: sterility control; GC: growth control. The bar graph shows the average inhibition diameters (mm) for both compounds. (b) Dose–response curve and IC_50_ value of the EtOAc extract. (c) Minimum inhibitory concentration (MIC) determination of the EtOAc extract by broth microdilution. (d) Dose–response curve and IC_50_ value of tetracycline (TET) and (e) MIC determination of tetracycline by broth microdilution. Red arrowheads indicate the MIC and the last well of visible turbidity.

The EtOAc crude extract obtained from the liquid culture of *A. chevalieri* showed inhibitory activity against *S.* Pullorum. The bioautography test revealed inhibition halos measuring 6.8 ± 0.3 mm on average, compared to tetracycline with an inhibition halo of 15.5 ± 0.5 mm (Figure 3a; Table S4). The MIC (4 mg/mL) and IC_50_ (3.00 ± 0.58 mg/mL) values of the EtOAc crude extract highlighted a moderate antimicrobial potency against *S.* Pullorum (Figure 3b,c). The reference antibiotic, tetracycline, had an IC_50_ of 0.097 ± 0.021* μ*g/mL and a MIC of 0.5 *μ*g/mL (Figure 3d,e).

### 3.3. Bioguided Fractionation of the EtOAc Crude Extract

**Figure 4:**
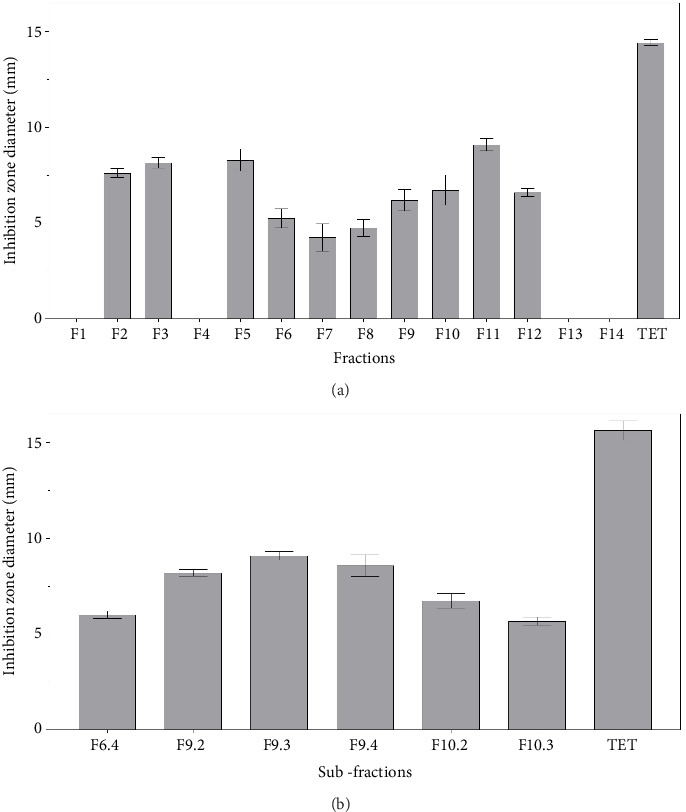
Bioautography and diameters of inhibition halos of active fractions obtained from (a) EtOAc crude extract and active subfractions obtained from (b) F6, F9, and F10. Reference drug: tetracycline (TET).

The EtOAc crude extract, separated by preparative liquid chromatography, yielded 14 fractions. Bioautography against *S.* Pullorum revealed antibacterial activity in 11 fractions (F2, F3, and F5–F12; Figure 4a; Figure S1). While Fractions F2, F3, F5, and F11 exhibited larger inhibition halos (>7.5 mm), Fractions F6, F9, and F10 were prioritized for further purification due to their higher mass yields (Table S5), which ensured sufficient material for bioguided fractionation and putative compound annotation. These three fractions showed moderate antimicrobial activity, with average inhibition zone diameters of 5.3 ± 0.5, 6.2 ± 0.6, and 6.7 ± 0.8 mm, respectively. Semipreparative chromatography of F6, F9, and F10 yielded several subfractions, among which F9.2 (23.3 mg), F9.3 (21.8 mg), F9.4 (18.7 mg), and F10.2 (13.1 mg) showed the highest antibacterial activity according to the bioautography test (Figure 4b; Table S7; Figure S2).

### 3.4. Annotation of Active Compounds

The most active subfractions (F9.2–9.4 and F10.2) were analyzed by UHPLC/HRMS in positive ionization mode. This analysis led to the putative annotation of 10 known compounds, based on their experimental mass, MS/MS fragmentation patterns, cosine similarity scores ≥ 0.7, and retention times. These annotations were established by spectral matching with publicly available GNPS databases (NIST, MoNa, and MassBank) and supported by previous literature reports (Table 3).

The putatively annotated compounds included two peptide derivatives ([1] cyclo(L-Tyr-L-Pro) and [2] *N*-acetyltyramine), a benzofuran derivative ([3] 1-[3-hydroxy-2-(2-hydroxy-2-propanyl)-2,3-dihydro-1-benzofuran-5-yl]ethanone), an aminopurine ([4] isopentenyladenine), a flavin ([5] lumichrome), an isocoumarin ([6] diaporthin), two indole diketopiperazines ([7] Neoechinulin A and [10] echinulin), and two diterpenoids ([8] 4-deoxyphorbol-13-acetate and [9] austinoneol). Apart from echinulin and Neoechinulin A, these compounds are reported for the first time from *A. chevalieri*. Chromatograms and mass spectrums of all compounds are presented in Figures S3–S11.

**Figure 5:**
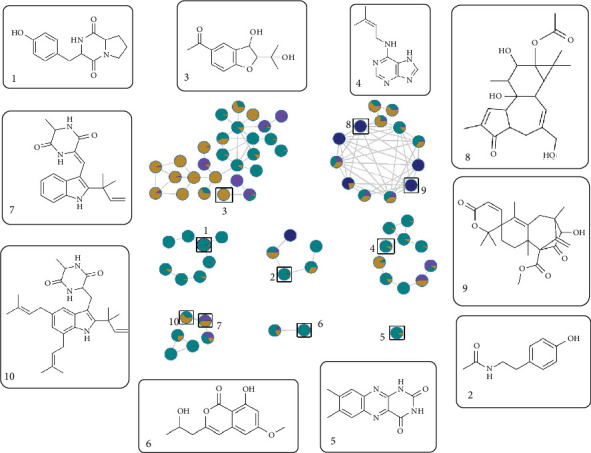
Molecular network of Subfractions 9.2, 9.3, 9.4, and 10.2, alongside the structures of putatively annotated compounds through GNPS database matching. Each node in the molecular network corresponds to a chemical compound and is visually represented as a pie chart. The pie chart illustrates the proportional distribution of the compound across the subfractions, color-coded as follows: 9.2 (purple), 9.3 (navy blue), 9.4 (teal), and 10.2 (dark gold). Chemical structures of (1) cyclo(L-Tyr-L-Pro); (2) *N*-acetyltyramine; (3) 1-[3-hydroxy-2-(2-hydroxy-2-propanyl)-2,3-dihydro-1-benzofuran-5-yl]ethanone; (4) isopentenyladenine; (5) lumichrome; (6) diaporthin; (7) Neoechinulin A; (8) 4-deoxyphorbol-13-acetate; (9) austinoneol; and (10) echinulin.

Additionally, a molecular network was generated using GNPS (Figure 5) (publicly accessible at https://gnps.ucsd.edu/ProteoSAFe/status.jsp?task=6a1534e08f4b409c8f3f78317d4c0cc8), which grouped the putative annotated compounds into clusters based on structural similarity. For instance, Compounds 7 and 10, both indole diketopiperazines, and Compounds 8 and 9, both diterpenes, were clustered together, highlighting shared biosynthetic pathways, while Compound 5 (a flavin) formed an isolated node, suggesting unique structural properties.

## 4. Discussion

### 4.1. Fungal Identification and Ecological Significance

This study represents the first report of a possible symbiotic association between *H. illucens* and *A. chevalieri*. The robust identification of *A. chevalieri*, using a combination of ITS, benA, and CaM markers, is consistent with current best practices in fungal taxonomy, due to resemblance to related species [55]. This finding aligns with other reports of fungi isolated from insect microbiomes, suggesting potential mutualistic relationships where fungi may contribute enzymes or antimicrobial metabolites that protect the insect from pathogens while benefiting from a nutrient-rich environment [56].

The marked growth promotion under high glucose (25%) and inhibition under high NaCl (15%) reflect the xerophilic and low-water-activity tolerance of *A. chevalieri*, consistent with its ecological adaptation to nutrient-rich but moisture-limited niches such as animal feed [18, 20, 22]. These results further support its classification within the *Aspergillus* section (formerly *Eurotium*), which includes species with pronounced osmophilic traits.

The presence of *A. chevalieri* in the gut microbiota of *H. illucens* larvae adds to our understanding of the complex microbial communities associated with this insect. Previous studies have shown that the gut microbiota of *H. illucens* larvae plays a crucial role in organic matter decomposition, nutrient cycling, and protection against pathogens [6, 7]. The identification of *A. chevalieri* as part of this microbiota suggests it may contribute to these functions, particularly in antimicrobial defense.

### 4.2. Antimicrobial Activity and Putative Annotation of Bioactive Compounds

The moderate antimicrobial activity of the *A. chevalieri* EtOAc crude extract against *S.* Pullorum (MIC = 4.00 mg/mL; IC_50_ = 3.00 ± 0.58 mg/mL) supports the antibacterial potential of fungal secondary metabolites [54, 57]. This finding demonstrates the potential of bioactive components from *A. chevalieri* for controlling poultry diseases and implies that the gut microbiota of *H. illucens* might have a protective ecological function by generating bioactive substances to offset microbial competition in the larval environment [58].

The bioguided fractionation approach effectively identified the most active fractions and facilitated the putative annotation of key bioactive compounds. This method has proven effective for isolating bioactive secondary metabolites from fungal extracts, particularly when dealing with complex mixtures [54]. The consistent activity observed across multiple fractions suggests either common biosynthetic routes or synergistic effects due to the presence of active metabolites distributed throughout the fractions [57].

The chemical diversity annotated in the active fractions reflects the rich metabolic potential of *A. chevalieri*. The 10 compounds putatively identified represent several structural classes with known biological activities.

#### 4.2.1. Indole Diketopiperazines

Two indole diketopiperazines, a chemical family commonly produced by *A. chevalieri* [19], were annotated in this study. Neoechinulin A (7) is commonly found in species of the section *Aspergillus*, including *A. chevalieri* [23], *A. amstelodami* [59], and *A. ruber* [49]. Previous research showed weak antibacterial effects against certain pathogens [49, 54], although one of its analogs, Neoechinulin B, showed high antibacterial activity against *Aeromonas hydrophila* (MIC = 4* μ*g/mL) and *E. coli* (MIC = 8* μ*g/mL) [23]. Neoechinulin A also exhibited antiviral potential against Influenza A virus and Herpes Simplex Virus Type 1 [54], inhibits the Mpro protease of SARS-CoV-2 (IC_50_ = 0.47* μ*M) [51], and has anticancer properties [60].

Echinulin (10), also annotated, is another compound commonly found in *Aspergillus* species [19, 49, 50, 54]. It has shown limited antibacterial activity against terrestrial bacteria [61, 62] but demonstrates antibacterial and algaecidal properties in marine environments [25]. Furthermore, echinulin was found to reduce melanin production in B16 melanoma cells (IC_50_ = 98.0 ± 1.2* μ*M), suggesting potential applications in the treatment of hyperpigmentation [63].

#### 4.2.2. Diterpenes

Two diterpenes, 4-deoxyphorbol-13-acetate (8) and austinoneol (9), were annotated in the active fractions. 4-Deoxyphorbol-13-acetate, a tetracyclic tigliane–type diterpene, has been reported as an insecticide against the red mite (*Dermanyssus gallinae*) [53], indicating its potential for controlling poultry ectoparasites. Notably, this is the first report of a phorbol derivative in *A. chevalieri*.

Although austinoneol—a meroterpene previously identified in *Aspergillus* and *Penicillium* species [64–66]—has not been directly linked to antimicrobial activity, its presence in the active Subfraction 9.3 implies a possible antibacterial effect against *S.* Pullorum that merits further investigation.

#### 4.2.3. Amino Acid Derivatives

Among the annotated metabolites were the amino acid derivatives cyclo(L-Tyr-L-Pro) (1) and *N*-acetyltyramine (2). Maculosin has demonstrated antiviral potential against the Hepatitis C virus by inhibiting the NS3 protease (IC_50_ = 8.2 ± 1.7* μ*g/mL) [24] alongside notable antioxidant activity [42]. *N*-Acetyltyramine possesses antibacterial activity against Gram-negative bacteria such as *Vibrio anguillarum* [43] and additionally functions as a quorum-sensing inhibitor in *Pseudomonas aeruginosa*, a key mechanism for bacterial virulence [67].

#### 4.2.4. Other Compounds

The remaining annotated metabolites comprised a benzofuran analog (3), structurally related to known antibacterial agents [46, 68]; isopentenyladenine (4), an aminopurine with reported antimicrobial properties [69]; lumichrome (5), flavin derivative exhibiting moderate antibacterial activity [44]; and diaporthin (6), an isocoumarin with antimicrobial activity against several pathogens, including *Escherichia coli*, *Micrococcus luteus*, MRSA, and *Staphylococcus aureus* [70].

### 4.3. Implications for Poultry Health and Antimicrobial Research

This study focused exclusively on *S.* Pullorum, given its particular importance in epidemiology and impact on the poultry industry. While many nontyphoidal *Salmonella* serovars can infect various hosts and usually result in self-limiting gastroenteritis in humans [8], *S.* Pullorum is avian-specific, causing a severe, often fatal, systemic infection in young chickens known as Pullorum disease [11].

Poultry flocks in low- and middle-income countries are especially vulnerable to this disease, which results in high mortality rates due to limited or nonexistent eradication programs. Due to decreased productivity, higher veterinary costs, and trade restrictions, the economic losses from Pullorum disease highlight the critical need for alternative, sustainable control strategies beyond conventional antibiotics [8, 13].

No additional *Salmonella* serovars were screened in this study. The investigation was purposely centered on *S*. Pullorum to assess the potential of fungal metabolites from insects in targeting poultry-specific pathogens. Further research could involve expanding the screening panel to include serovars like *S*. Typhimurium or *S*. Enteritidis to explore the full range of antimicrobial activity of the compounds found.

The identification of multiple bioactive compounds from *A. chevalieri* with potential activity against *S.* Pullorum has significant implications for poultry health and antimicrobial research. Pullorum disease remains a concern in many parts of the world, particularly in developing countries where biosecurity measures may be less stringent [11]. The discovery of novel antimicrobial compounds from natural sources like insect gut microbiota represents a promising approach to address the challenges of antibiotic resistance in poultry production [16, 17].

Furthermore, this research highlights the potential of insect microbiota as a source of bioactive compounds with applications beyond poultry health. The diverse chemical structures and biological activities of the identified compounds suggest potential applications in human medicine, veterinary science, and agricultural pest management.

### 4.4. Limitations and Future Directions

While this study provides valuable insights into the antimicrobial potential of *A. chevalieri* from *H. illucens* gut microbiota, several limitations should be acknowledged. First, the identification of compounds was based on UHPLC/HRMS analysis and spectral database comparisons, which provide putative annotations rather than definitive identifications. Future studies should focus on the isolation and structural confirmation of these compounds through nuclear magnetic resonance (NMR) spectroscopy and other analytical techniques.

Second, the antimicrobial activity was evaluated against a single pathogen, *S.* Pullorum. Expanding the screening to include other poultry pathogens and antibiotic-resistant strains would provide a more comprehensive assessment of the antimicrobial potential of these compounds.

Third, the mechanisms of action of these compounds against *S.* Pullorum remain unknown. Studies investigating the cellular targets and molecular mechanisms would enhance our understanding of their antimicrobial properties and potential for development as therapeutic agents.

Future research should focus on isolation and structural confirmation of the putative annotated compounds, evaluation of their antimicrobial activity against a broader range of pathogens, investigation of the mechanisms of action against *S.* Pullorum, assessment of their efficacy in in vivo models of pullorum disease, and exploration of potential synergistic effects between different compounds.

## 5. Conclusion

This study demonstrates that *A. chevalieri* isolated from the gut microbiota of *H. illucens* larvae produces bioactive compounds with antimicrobial activity against *S.* Pullorum. Ten compounds with diverse chemical structures and potential antimicrobial properties were putatively annotated, including indole diketopiperazines, peptide derivatives, a benzofuran derivative, an isocoumarin, a flavin, an aminopurine, and two diterpenoids. This represents the first report of *A. chevalieri* associated with *H. illucens* larvae and highlights the potential of insect gut microbiota as a source of novel antimicrobial compounds.

The putative annotation of these bioactive compounds provides a foundation for the development of new antimicrobial agents for poultry health, potentially addressing the challenges of antibiotic resistance in the poultry industry. Further research on the isolation, structural confirmation, and mechanisms of action of these compounds will enhance our understanding of their antimicrobial properties and potential therapeutic applications.

## Figures and Tables

**Table 1:** Primer sequences used for fungal isolate identification.

**Molecular marker**	**Primer name**	**Primer sequence (5**⁣′**–3**⁣′**)**	**Product length**
*β*-Tubulin (benA)	bt2a F	GGTAACCAAATCGGTGCTGCTTTC	440
	bt2b R	ACCCTCAGTGTAGTGACCCTTGGC	
Calmodulin (CaM)	cmd5 F	CCGAGTACAAGGAGGCCTTC	548
	cmd6 R	CCGATAGAGGTCATAACGTGG	
ITS	ITS5 F	GGAAGTAAAAGTCGTAACAAGG	487
	ITS4 R	TCCTCCGCTTATTGATATGC	

**Table 2:** Accession numbers of amplified nucleotide sequences from the HGU11_3 isolate.

**Identification**	**Calmodulin (CaM)**	***β*-Tubulin (benA)**	**ITS**
*A. chevalieri*	PP861110	PP861111	PP860889

**Table 3:** Characterization of the chemical constituents of *A. chevalieri* in Subfractions F9.2, F9.3, F9.4, and F10.2.

**No.**	**Putative ID**	**Cosine**	**RT (min)**	**Experimental mass**(**m**/**z**)	**MS/MS fragments**	**Mass error (ppm)**	**Formula**	**Fraction**	**Ref.**
1	Cyclo(L-Tyr-L-Pro)	0.83	5.90	261.1234^a^	70.0658, 98.0605, 107.0495, 136.0758, 155.0815	0.53	C_14_H_16_N_2_O_3_	9.4	[40–42]
2	*N*-acetyltyramine	0.97	7.14	180.1018^a^	121.0651, 180.1018	0.47	C_10_H_13_NO_2_	9.4	[43, 44]
3	1-[3-Hydroxy-2-(2-hydroxy-2-propanyl)-2,3-dihydro-1-benzofuran-5-yl]ethanone	0.78	8.93	237.0693^a^	165.0482, 219.05, 237.0693	1.69	C_13_H_16_O_4_	10.2	[45]
4	Isopentenyladenine	0.99	9.11	204.1236^a^	69.0706, 136.0619, 148.0618	0.38	C_10_H_13_N_5_	9.4	[46]
5	Lumichrome	0.99	11.59	243.0876^a^	172.0868, 198.0660, 216.0767	0.36	C_12_H_10_N_4_O_2_	9.4	[44, 45]
6	Diaporthin	0.77	13.76	251.0914^a^	135.0442, 177.0548, 191.0339, 205.0497, 233.0809, 251.0889	0.08	C_13_H_14_O_5_	9.4	[46–48]
7	Neoechinulin A	0.81	14.68	324.1339^a^	69.0705, 157.0760, 185.0708, 256.0714, 268.0715	1.20	C_19_H_21_N_3_O_2_	9.2; 10.2	[24, 49–51]
8	4-Deoxyphorbol-13-acetate	0.89	17.66	432.2378^b^	107.0859, 119.0858, 127.0387, 135.0805	0.33	C_22_H_30_O_6_	9.3	[52, 53]
9	Austinoneol	0.97	17.66	415.2111^a^	119.0857	0.52	C_24_H_30_O_6_	9.3	[54]
10	Echinulin	0.89	20.99	462.3109^a^	69.0705, 198.1277, 211.1354, 266.1902, 338.1868, 406.2488	1.16	C_29_H_39_N_3_O_2_	9.4	[49, 50]

*Note:* Cosine scores reflect the degree of MS/MS spectral similarity; values ≥0.7 significantly increase confidence in the match.

^a^Corresponds to the adduct [M + H]^+^.

^b^Corresponds to the adduct [M + ACN + H]^+^.

## Data Availability

The data presented in this study are available upon request from the corresponding author.

## Supporting Information

Supporting Information Additional supporting information can be found online in the Supporting Information section. Table S1. Sequences with significant alignments of the calmodulin gene (CaM) for the HGU11_3 isolate. Table S2. Sequences with significant alignments of the *β*-tubulin gene (benA) for the HGU11_3 isolate. Table S3. Sequences with significant alignments of the ITS region for the HGU11_3 isolate. Table S4. Inhibition halo diameter averages (mm) from bioautography test of Fractions F2, F3, and F5 to F12 and TET. Table S5. Total masses (mg) of the fractions obtained from the EtOAc crude extract. Table S6. Total masses (mg) of the subfractions obtained from Fractions 6, 9, and 10. Table S7. Inhibition halo diameter averages (mm) from bioautography test of Subfractions 6, 9, and 10 and TET. Figure S1. Bioautography of fractions from EtOAc crude extract of *Aspergillus chevalieri.* Figure S2. Bioautography of the subfractions obtained from Fractions F6, F9, and F10. Figure S3. High-performance liquid chromatography (HPLC) chromatogram of Fraction 9.2 from *A. chevarieri* EtOAc crude extract. Figure S4. Mass spectrometry (HESI-MS/MS) spectrum of Compound 7 (Neoechinulin A). Figure S5. High-performance liquid chromatography (HPLC) chromatogram of Fraction 9.3 from *A. chevarieri* EtOAc crude extract. Figure S6. Mass spectrometry (HESI-MS/MS) spectra of diterpenoids: Compounds 8 (4-deoxyphorbol-13-acetate) and 9 (austinoneol). Figure S7. High-performance liquid chromatography (HPLC) chromatogram of Fraction 9.4 from *A. chevarieri* EtOAc crude extract. Figure S8. Mass spectrometry (HESI-MS/MS) spectra of peptide derivatives: Compounds 1 (cyclo(L-Tyr-L-Pro)) and 2 (*N*-acetyltyramine). Figure S9. Mass spectrometry (HESI-MS/MS) spectra of Compound 4 (isopentenyladenine), Compound 5 (lumichrome), Compound 6 (diaporthin), and Compound 10 (echinulin). Figure S10. High-performance liquid chromatography (HPLC) chromatogram of Fraction 10.2 from *A. chevalieri* EtOAc crude extract. Figure S11. Mass spectrometry (HESI-MS/MS) spectra of Compound 3 (1-[3-Hydroxy-2-(2-hydroxy-2-propanyl)-2,3-dihydro-1-benzofuran-5-yl] ethanone).
