# Supplementary material for: Antimicrobial Compounds From Aspergillus chevalieri Associated With the Gut Microbiota of Hermetia illucens Larvae Targeting Salmonella enterica Serovar Pullorum
Source: Int J Microbiol. 2025 Oct 15;2025:8756981. doi: 10.1155/ijm/8756981 (PMC12543498; doi:10.1155/ijm/8756981)
Supplement: Supporting Information — Additional supporting information can be found online in the Supporting Information section. Table S1. Sequences with significant alignments of the calmodulin gene (CaM) for the HGU11_3 isolate. Table S2. Sequences with significant alignments of the β-tubulin gene (benA) for the HGU11_3 isolate. Table S3. Sequences with significant alignments of the ITS region for the HGU11_3 isolate. Table S4. Inhibition halo diameter averages (mm) from bioautography test of Fractions F2, F3, and F5 to F12 and TET. Table S5. Total masses (mg) of the fractions obtained from the EtOAc crude extract. Table S6. Total masses (mg) of the subfractions obtained from Fractions 6, 9, and 10. Table S7. Inhibition halo diameter averages (mm) from bioautography test of Subfractions 6, 9, and 10 and TET. Figure S1. Bioautography of fractions from EtOAc crude extract of Aspergillus chevalieri. Figure S2. Bioautography of the subfractions obtained from Fractions F6, F9, and F10. Figure S3. High-performance liquid chromatography (HPLC) chromatogram of Fraction 9.2 from A. chevarieri EtOAc crude extract. Figure S4. Mass spectrometry (HESI-MS/MS) spectrum of Compound 7 (Neoechinulin A). Figure S5. High-performance liquid chromatography (HPLC) chromatogram of Fraction 9.3 from A. chevarieri EtOAc crude extract. Figure S6. Mass spectrometry (HESI-MS/MS) spectra of diterpenoids: Compounds 8 (4-deoxyphorbol-13-acetate) and 9 (austinoneol). Figure S7. High-performance liquid chromatography (HPLC) chromatogram of Fraction 9.4 from A. chevarieri EtOAc crude extract. Figure S8. Mass spectrometry (HESI-MS/MS) spectra of peptide derivatives: Compounds 1 (cyclo(L-Tyr-L-Pro)) and 2 (N-acetyltyramine). Figure S9. Mass spectrometry (HESI-MS/MS) spectra of Compound 4 (isopentenyladenine), Compound 5 (lumichrome), Compound 6 (diaporthin), and Compound 10 (echinulin). Figure S10. High-performance liquid chromatography (HPLC) chromatogram of Fraction 10.2 from A. chevalieri EtOAc crude extract. Figure S11. Mass spectr [file 8756981.f1.pdf]

Supplementary Material

Table S1. Sequences with significant alignments of the calmodulin gene (CaM) for the HGU11_3 isolate.

	Description	Query Cover	E value	Per. Ident
MK451334.1	*Aspergillus chevalieri* strain CMV011B7 calmodulin gene, partial cds	100%	0	100%
MK451333.1	*Aspergillus chevalieri* strain CMV011B6 calmodulin gene, partial cds	100%	0	100%
MK451332.1	*Aspergillus chevalieri* strain CMV003I3 calmodulin gene, partial cds	100%	0	100%
MK951911.1	*Aspergillus chevalieri* strain CMV016D7 calmodulin gene, partial cds	100%	0	100%
LC494259.1	*Aspergillus chevalieri* M2 CaM gene for calmodulin, partial cds	100%	0	100%

Table S2. Sequences with significant alignments of the β-tubulin gene (benA) for the HGU11_3 isolate.

Accession	Description	Query Cover	E value	Per. Ident
KX455755.1	*Aspergillus chevalieri* isolate VPCI 449/P/14 beta-tubulin (BT) gene, partial cds	99%	0	100.00%
KU872178.1	*Aspergillus chevalieri* strain TGN9 beta-tubulin (B-tub) gene, partial cds	99%	0	100.00%
LC733669.1	*Aspergillus chevalieri* Egy2-EU2 tub2 gene for beta-tubulin, partial cds	99%	0	100.00%
LC733668.1	*Aspergillus chevalieri* Egy1-EU1 tub2 gene for beta-tubulin, partial cds	99%	0	100.00%
MZ027912.1	*Aspergillus chevalieri* strain DTO 438-B1 beta-tubulin (benA) gene, partial cds	99%	0	100.00%

Table S3. Sequences with significant alignments of the ITS region for the HGU11_3 isolate.

Accession	Description	Query Cover	E value	Per. Ident
NR_135340.1	*Aspergillus chevalieri* NRRL 78 ITS region	100%	0	100.00%
NR_137448.1	*Aspergillus intermedius* NRRL 82 ITS region	100%	0	99.79%
NR_135434.1	*Aspergillus costiformis* CBS 101749 ITS region	99%	0	99.79%
NR_163674.1	*Aspergillus heterocaryoticus* CBS 410.65 ITS region	97%	0	100.00%
NR_135336.1	*Aspergillus pseudoglaucus* NRRL 40 ITS region	100%	0	98.97%

Table S4. Inhibition halo diameter averages (mm) from bioautography test of fractions F2, F3, and F5 to F12, and TET.

Fraction	Average (mm)
F2	7.6 ± 0.2
F3	8.2 ± 0.3
F5	8.3 ± 0.6
F6	5.3 ± 0.5
F7	4.3 ± 0.7
F8	4.8 ± 0.4
F9	6.2 ± 0.6
F10	6.7 ± 0.8
F11	9.1 ± 0.3
F12	6.6 ± 0.2
TET	14.4 ± 0.2

Table S5. Total masses (mg) of fractions obtained from EtOAc crude extract.

Fraction	Mass (mg)
1	34.6
2	4.9
3	15.3
4	7.2
5	5
6	177.5
7	61.7
8	48.9
9	316.3
10	149.4
11	55.1
12	133.5
13	504.4
14	60.4

Table S6. Total masses (mg) of sub-fractions obtained from fractions 6, 9, and 10.

Sub-Fraction	Mass	Sub-Fraction	Mass	Sub-Fraction	Mass
6.1	11.5	9.1	4.1	10.1	4.3
6.2	9.3	9.2	23.3	10.2	13.1
6.3	8	9.3	21.8	10.3	19.2
6.4	30.9	9.4	18.7	10.4	9.8
6.5	95.4	9.5	31.9	10.5	17.6
6.6	4	9.6	52.7	10.6	16.4
6.7	7	9.7	25.2	10.7	46.5
6.8	9	9.8	51.9	10.8	2.1
6.9	2.9	9.9	22.1	10.9	13.2
6.1	4.3	9.1	15.4		
6.11	5.9	9.11	8.5		
		9.12	11		
		9.13	8.9		
		9.14	8.2		
		9.15	11.9		

Table S7. Inhibition halo diameter averages (mm) from bioautography test of sub-fractions 6, 9, and 10, and TET.

Sub-Fraction	Average (mm)
F6.4	6.2 ± 0.2
F9.2	8.2 ± 0.2
F9.3	9.1 ± 0.2
F9.4	8.6 ± 0.6
F10.2	6.8 ± 0.4
F10.3	5.7 ± 0.2
TET	15.7 ± 0.5

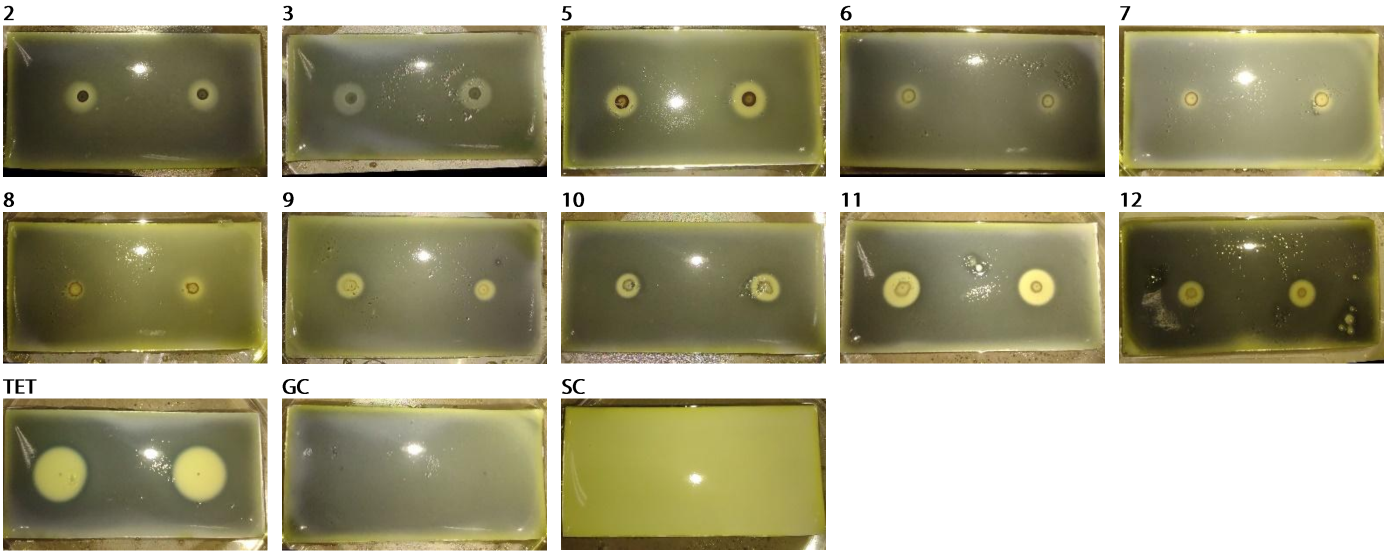

Figure S1. Bioautography of fractions from EtOAc crude extract of *Aspergillus chevalieri*. **2-12:** Active fractions of EtOAc crude extract; **TET:** reference drug (tetracycline); **GC:** growth control; **SC:** sterility control.

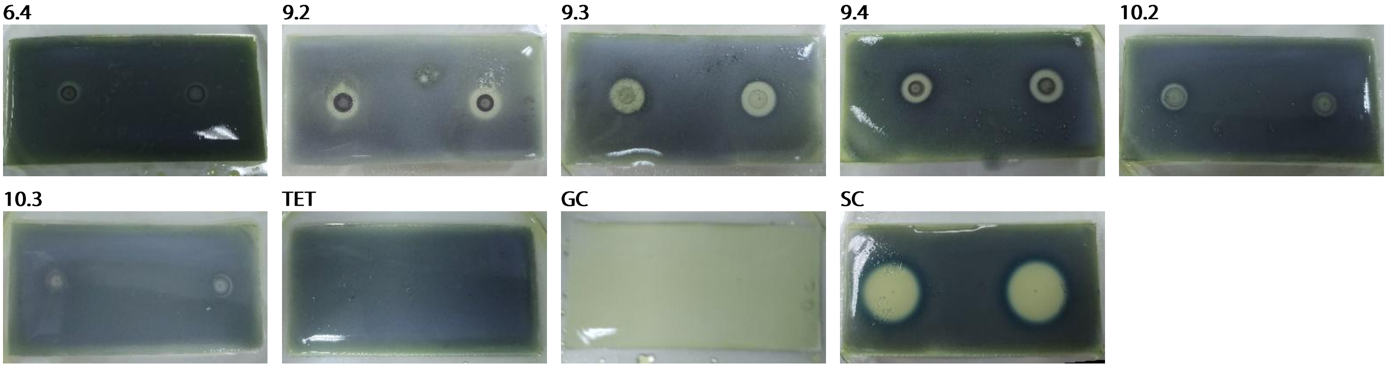

Figure S2. Bioautography of sub-fractions obtained from fractions F6, F9, and F10. **6.4, 9.2, 9.3, 9.4, 10.2,** and **10.3:** Active sub-fractions; **TET:** reference drug (tetracycline); **GC:** growth control; **SC:** sterility control.

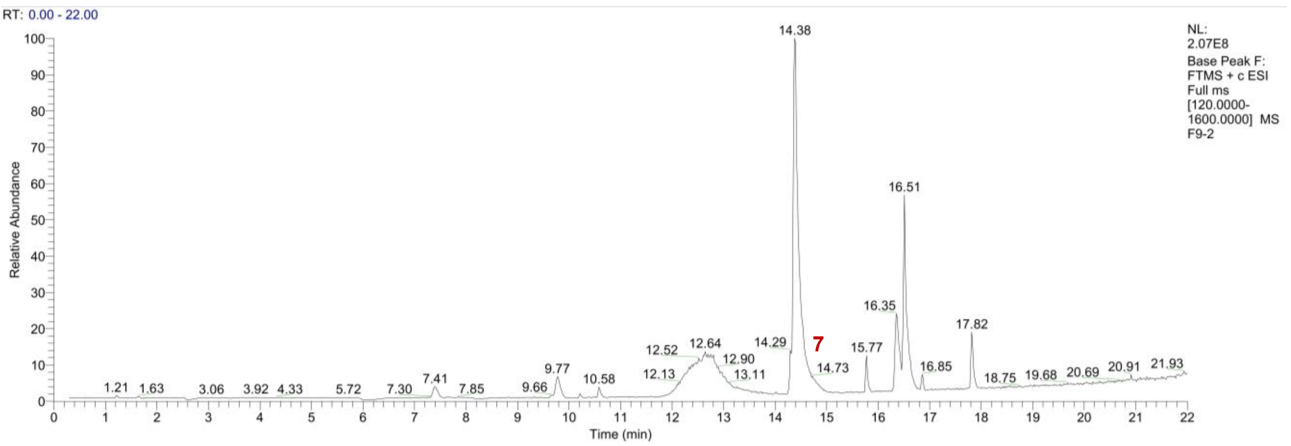

Figure S3. High Performance Liquid Chromatography (HPLC) chromatogram of fraction 9.2 from *A. chevarieri* EtOAc crude extract in a Luna Omega C-18 column Luna Omega (150 × 2.1 mm, 1.6 µm; Phenomenex, Torrance, CA, USA) using an UltiMate™ 3000 UHPLC system coupled with a Q Exactive Plus mass spectrometer (Thermo Scientific, Dreieich, Germany) in a mass range from 120 to 1600. The peak numbers in this figure to those used in Table 2.

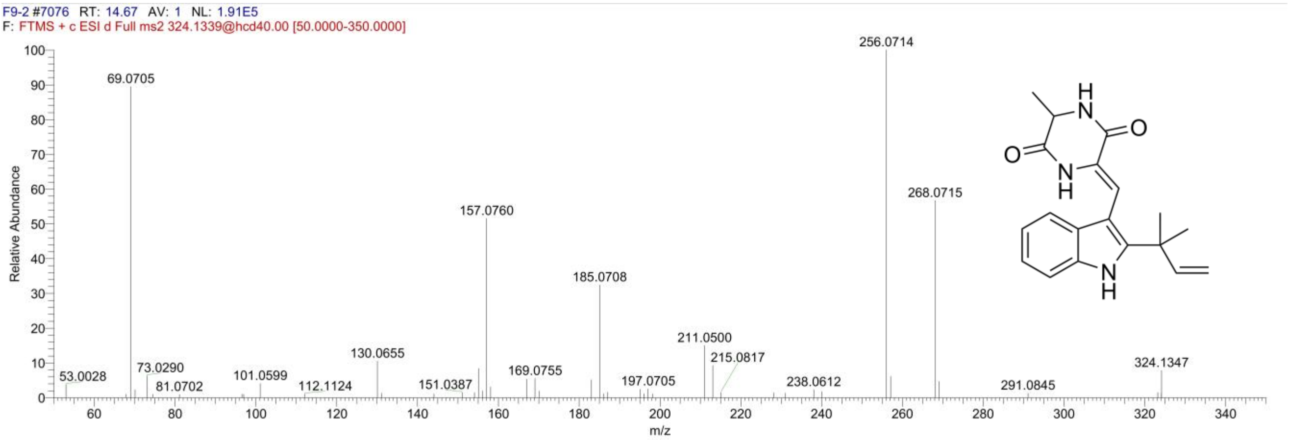

Figure S4. Mass spectrometry (HESI-MS/MS) spectrum of Compound 7, tentatively annotated as (3S,6Z)-3-methyl-6-[[2-(2-methylbut-3-en-2-yl)-1H-indol-3-yl]methylidene]piperazine-2,5-dione (neoechinulin A) an indole diketopiperazine, isolated from the EtOAc crude extract of *A. chevalieri* HGU11_3. The spectrum shows characteristic fragment ions consistent with those reported for neoechinulin A, supporting its putative annotation. The molecular structure of the compound is displayed on the right.

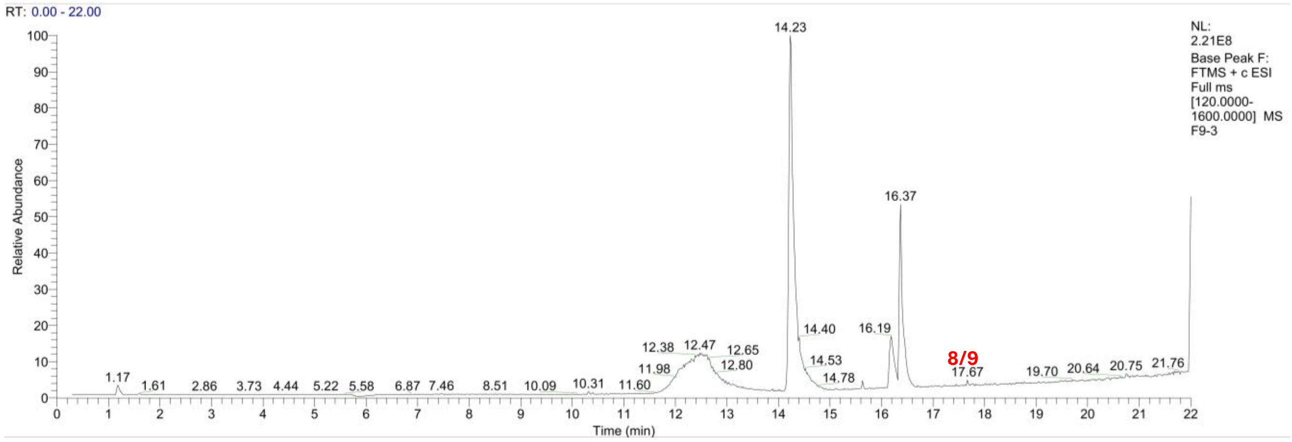

Figure S5. High Performance Liquid Chromatography (HPLC) chromatogram of fraction 9.3 from *A. chevarieri* EtOAc crude extract in a Luna Omega C-18 column Luna Omega (150 × 2.1 mm, 1.6 µm; Phenomenex, Torrance, CA, USA) using an UltiMate™ 3000 UHPLC system coupled with a Q Exactive Plus mass spectrometer (Thermo Scientific, Dreieich, Germany) in a mass range from 120 to 1600. The peak numbers in this figure to those used in Table 2.

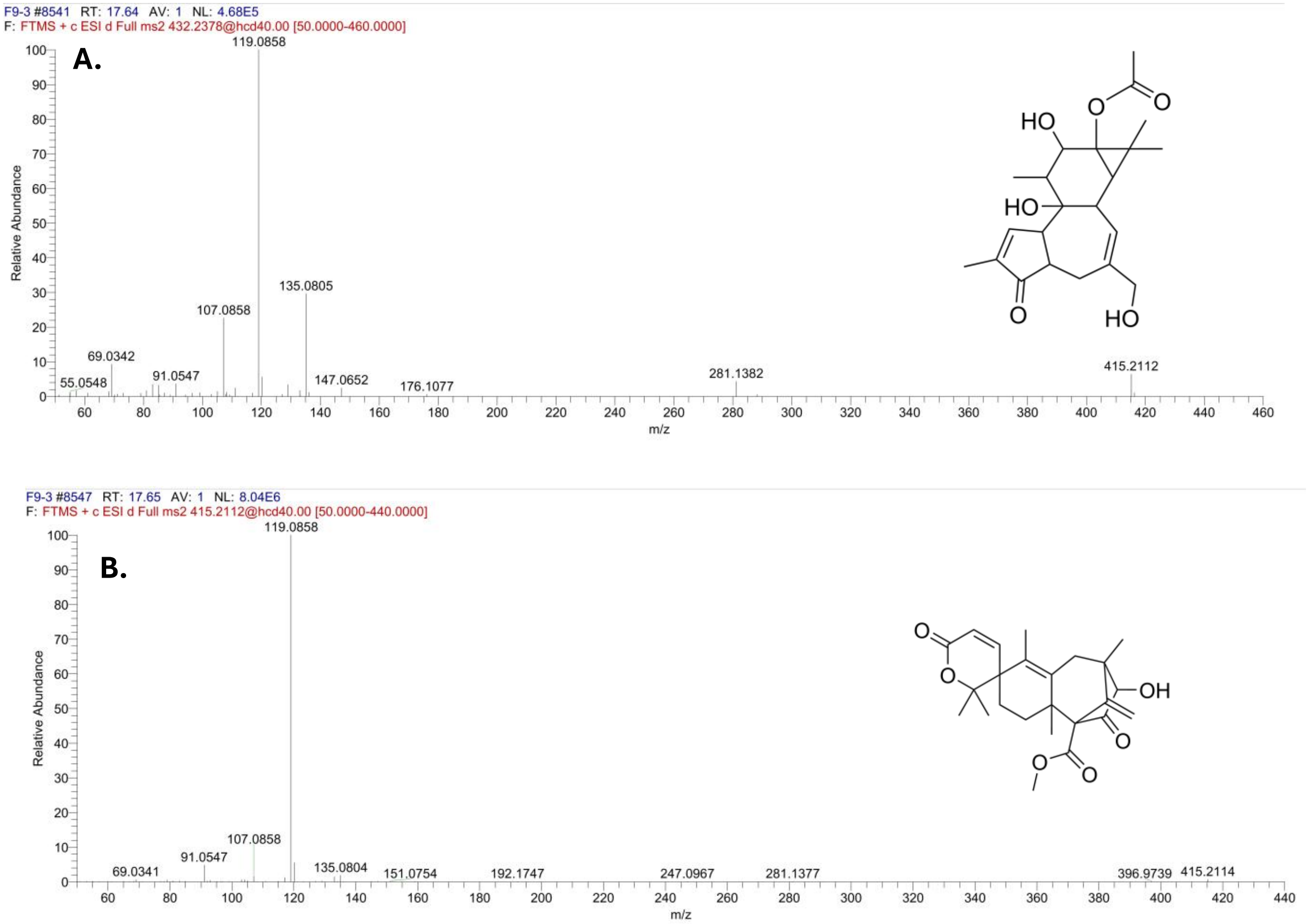

Figure S6. Mass spectrometry (HESI-MS/MS) spectra of diterpenoids tentatively annotated from the ethyl acetate crude extract of *A. chevalieri* HGU11_3. A. Spectrum of Compound 8, tentatively annotated as 7b,9-dihydroxy-3-(hydroxymethyl)-1,1,6,8-tetramethyl-5-oxo-1,1a,1b,4,4a,5,7a,7b,8,9-decahydro-9aH-cyclopropa[3,4]benzo[1,2-e]azulen-9a-yl acetate (4-Deoxyphorbol-13-acetate). B. Spectrum of Compound 9 tentatively annotated as Austinoneol. The spectrum shows characteristic fragment ions consistent with those reported for both diterpenes, supporting its putative annotation, with their molecular structures shown on the right.

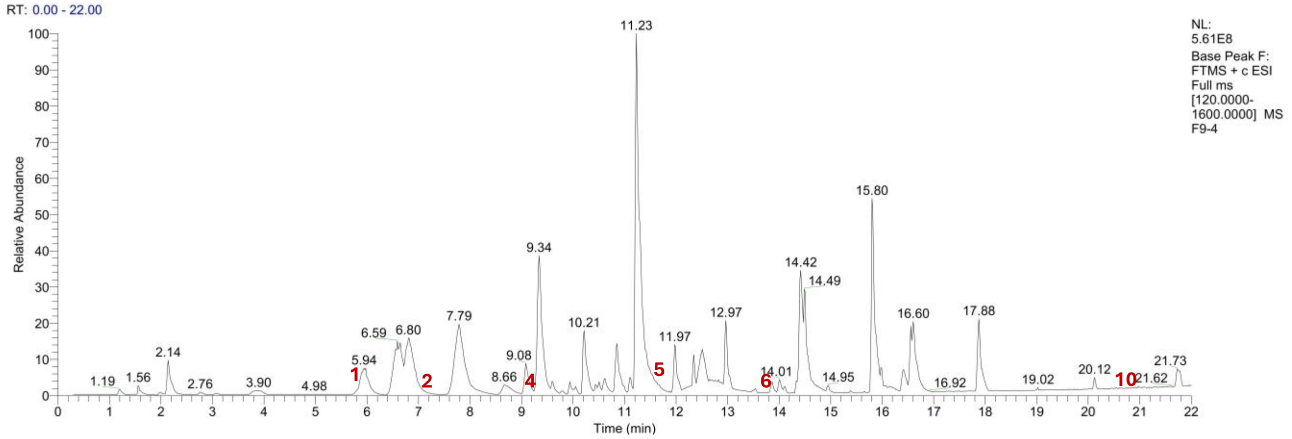

Figure S7. High Performance Liquid Chromatography (HPLC) chromatogram of fraction 9.4 from *A. chevarieri* EtOAc crude extract in a Luna Omega C-18 column Luna Omega (150 × 2.1 mm, 1.6 µm; Phenomenex, Torrance, CA, USA) using an UltiMate™ 3000 UHPLC system coupled with a Q Exactive Plus mass spectrometer (Thermo Scientific, Dreieich, Germany) in a mass range from 120 to 1600. The peak numbers in this figure to those used in Table 2

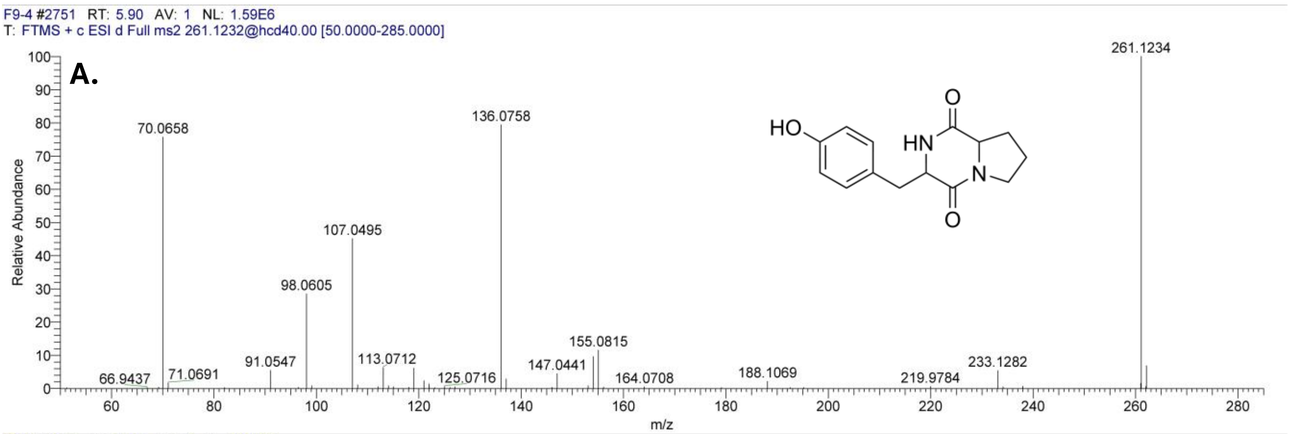

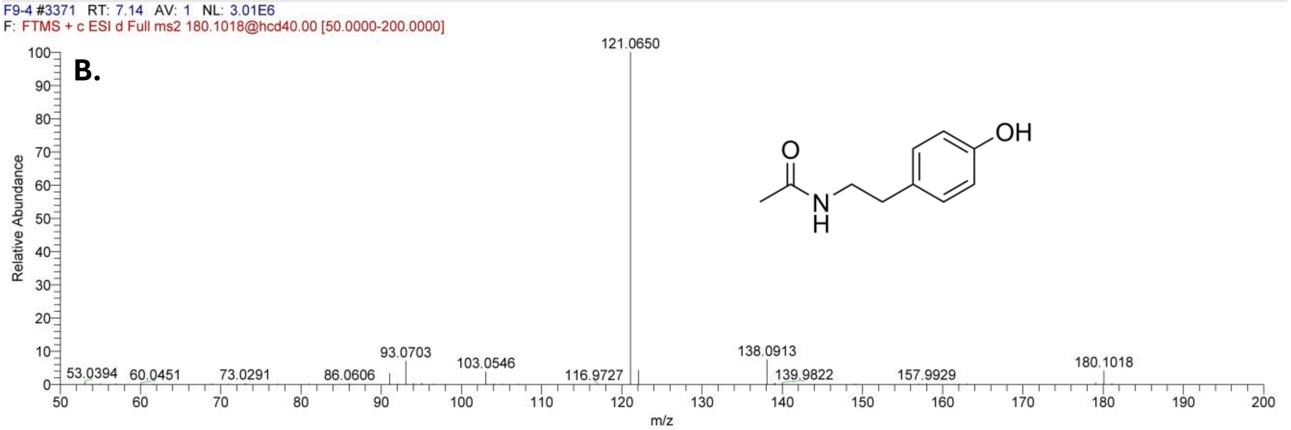

Figure S8. Mass spectrometry (HESI-MS/MS) spectra of peptide derivatives tentatively annotated from the EtOAc crude extract of *Aspergillus chevalieri* HGU11_3. **A.** Spectrum of Compound 1, tentatively annotated as cyclo(L-Tyr-L-Pro), a cyclic dipeptide belonging to the diketopiperazine class. **B.** Spectrum of Compound 2, tentatively annotated as *N*-acetyltyramine, a peptide derivative. The spectrum shows characteristic fragment ions consistent with those reported for both peptide derivatives, supporting its putative annotation, with their molecular structures depicted on the right.

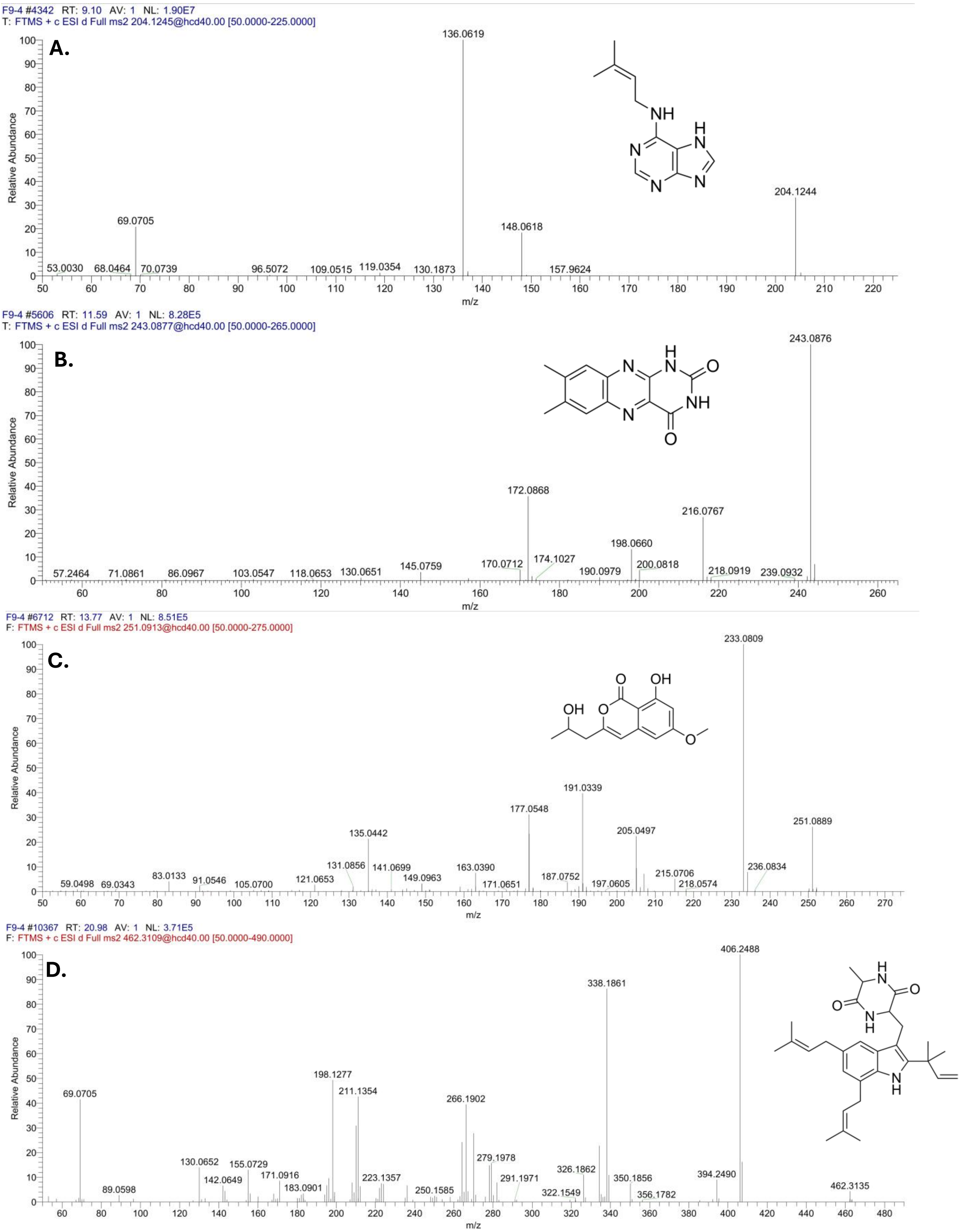

Figure S9. Mass spectrometry (HESI-MS/MS) spectra of additional compounds tentatively annotated from the EtOAc crude extract of *A. chevalieri* HGU11_3. **A.** Spectrum of Compound 4, tentatively annotated as Isopentenyladenine, an aminopurine derivative. **B.** Spectrum of Compound 5, tentatively annotated as Lumichrome, a compound belonging to the flavin class. **C.** Spectrum of Compound 6, tentatively annotated as Diaporthin, classified as an isocoumarin. **D.** Spectrum of Compound 10, tentatively annotated as Echinulin, an indole diketopiperazine. The spectrum shows characteristic fragment ions consistent with those reported for these structurally diverse metabolites, supporting its putative annotation, with their molecular structures depicted on the right.

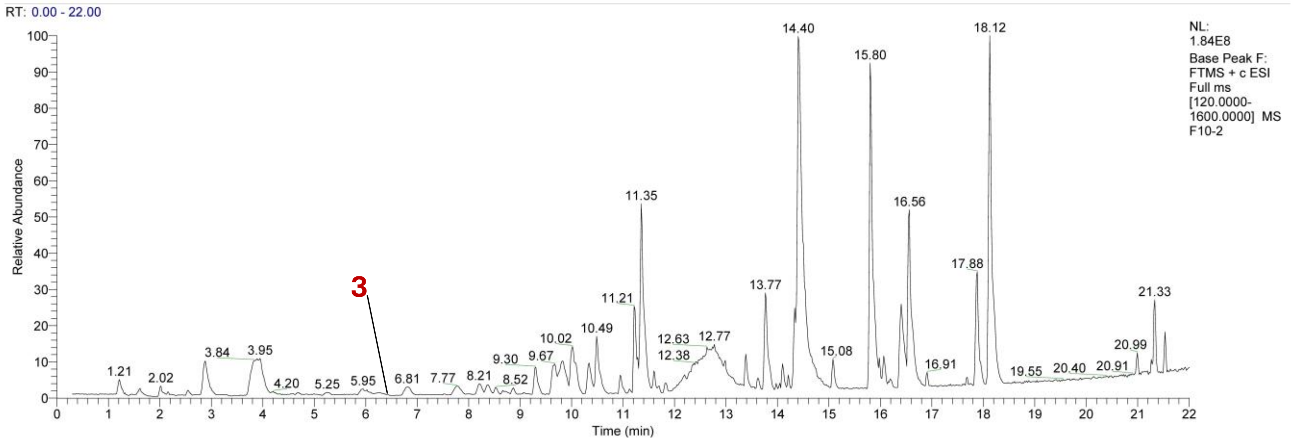

Figure S10. High Performance Liquid Chromatography (HPLC) chromatogram of fraction 10.2 from *A. chevarieri* EtOAc crude extract in a Luna Omega C-18 column Luna Omega (150 × 2.1 mm, 1.6 µm; Phenomenex, Torrance, CA, USA) using an UltiMate™ 3000 UHPLC system coupled with a Q Exactive Plus mass spectrometer (Thermo Scientific, Dreieich, Germany) in a mass range from 120 to 1600. The peak numbers in this figure to those used in Table 2.

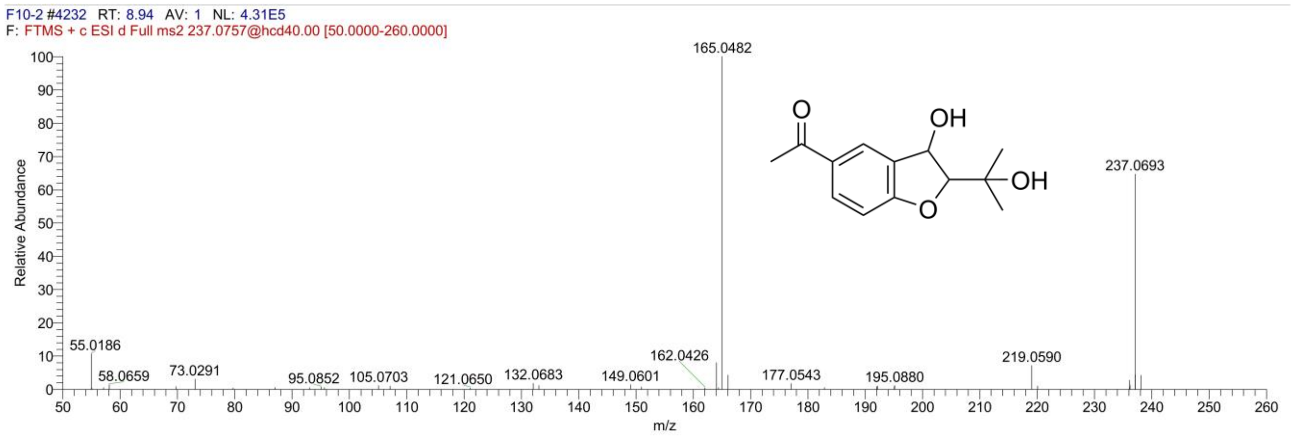

Figure S11. Mass spectrometry (HESI-MS/MS) spectra of Compound 3, tentatively annotated as 1-[3-Hydroxy-2-(2-hydroxy-2-propanyl)-2,3-dihydro-1-benzofuran-5-yl] ethanone, a benzofuran derivative. The spectrum shows characteristic fragment ions consistent with those reported for Compound 3, supporting its putative annotation. The molecular structure of the compound is displayed on the right.
